# Combination of *panax ginseng* and *ginkgo biloba* extracts attenuate cerebral ischemia injury with modulation of NLRP3 inflammasome and CAMK4/CREB pathway

**DOI:** 10.3389/fphar.2022.980449

**Published:** 2022-08-25

**Authors:** Aimei Zhao, Nan Liu, Guozhi Jiang, Li Xu, Mingjiang Yao, Yehao Zhang, Bingjie Xue, Bo Ma, Dennis Chang, Yujing Feng, Yunyao Jiang, Jianxun Liu, Guoping Zhou

**Affiliations:** ^1^ Department of Acupuncture and Moxibustion, Neuroscience Centre, Integrated Hospital of Traditional Chinese Medicine, Southern Medical University, Guangzhou, China; ^2^ Beijing Key Laboratory of Pharmacology of Chinese Materia, Institute of Basic Medical Sciences, Xiyuan Hospital of China Academy of Chinese Medical Sciences, Beijing, China; ^3^ Beijing Increasepharm Safety and Efficacy Co. Ltd., Beijing, China; ^4^ Shineway Pharmaceutical Group Co. Ltd., Shijiazhuang, China; ^5^ NICM, Western Sydney University, Penrith, NSW, Australia; ^6^ Department of Anesthesiology, Punan Hospital, Shanghai, China; ^7^ School of Pharmaceutical Sciences, Institute for Chinese Materia Medica, Tsinghua University, Beijing, China

**Keywords:** ischemic stroke, ginseng extract, ginkgo biloba extract, NLRP3 inflammasome, CAMK4/CREB, network analysis

## Abstract

Stroke is a major cause of death and disability throughout the world. A combination of *Panax Ginseng* and *Ginkgo biloba* extracts (CGGE) is an effective treatment for nervous system diseases, but the neuroprotective mechanism underlying CGGE remains unclear. Both network analysis and experimental research were employed to explore the potential mechanism of CGGE in treating ischemic stroke (IS). Network analysis identified a total number of 133 potential targets for 34 active ingredients and 239 IS-related targets. What’s more, several processes that might involve the regulation of CGGE against IS were identified, including long-term potentiation, cAMP signaling pathway, neurotrophin signaling pathway, and Nod-like receptor signaling pathway. Our studies in animal models suggested that CGGE could reduce inflammatory response by inhibiting the activity of Nod-like receptor, pyrin containing 3 (NLRP3) inflammasome, and maintain the balance of glutamate (Glu)/gamma-aminobutyric acid (GABA) via activating calmodulin-dependent protein kinase type Ⅳ (CAMK4)/cyclic AMP-responsive element-binding protein (CREB) pathway. These findings indicated the neuroprotective effects of CGGE, possibly improving neuroinflammation and excitotoxicity by regulating the NLRP3 inflammasome and CAMK4/CREB pathway.

## 1 Introduction

Stroke is one of the leading causes of mortality and disability, especially in low-income and middle-income countries (O'Donnell et al., 2016). According to the recent Global Burden of Disease survey, stroke has risen to the third position from the fifth (Collaborators, 2020). Approximately 87% of all strokes are ischemic strokes (Virani et al., 2020).

The underlying mechanism of ischemic stroke (IS) is complex. Excitotoxicity and inflammation play crucial roles in the pathophysiological process of IS. Overactivation of the glutamate receptor mediates excitotoxicity, which has been suggested as a critical event in ischemic brain injury. Glutamate (Glu) is a major excitatory neurotransmitter in the central nervous system and acts on metabotropic and ionotropic receptors (including N-methyl-D-aspartate receptor, NMDAR) to mediate excitatory synapse transmission (Traynelis et al., 2010). While gamma-aminobutyric acid (GABA) mainly works as an inhibitory neurotransmitter in the brain and modulates excitatory neurotransmission (Boddum et al., 2016). Under ischemic conditions, excessive glutamate over-stimulates NMDAR, causing massive Ca^2+^ influx. The increased intracellular Ca^2+^ causes mitochondrial dysfunction and overproduction of reactive oxygen species (ROS), which activates inflammatory responses and results in the death of damaged neurons (Zhou et al., 2018). NMDAR activation at a moderate level promotes neuroprotective signaling pathways, including activation of the RAS-mitogen-activated protein kinase (RAS-MAPK) pathway and cyclic AMP-responsive element-binding protein (CREB)-dependent gene transcription. Brain-derived neurotrophic factor (BDNF) plays a key role in neuronal development and synaptic plasticity, which could be promoted by CREB (Park and Poo, 2013). Tat-NR2B9c, a blocker of NMDAR-postsynaptic density protein-95 (PSD95), exerts a neuroprotective effect on stroke by activating Ca^2+^ dependent pathways and enhancing calmodulin-dependent protein kinase type Ⅳ (CaMKⅣ) and CREB (Bell et al., 2013). Nod-like receptor, pyrin containing 3 (NLRP3)-inflammasome is a member of innate cell sensors, which can produce a variety of proinflammatory cytokines and mediate nerve cell dysfunction, thus leading to cell death after cerebral ischemia (Xu et al., 2021). There is evidence of increased expression and activation of NLRP3 inflammasome in ischemic stroke neurons (Savage et al., 2012; Fann et al., 2018). The NLRP3 inflammasome is composed of three components, a sensor (NLRP3), an adaptor (ASC; apoptosis-associated speck-like protein containing a caspase recruitment domain) and an effector (caspase-1) (Swanson et al., 2019).

*Panax Ginseng* (*P.ginseng* C.A.Mey.) is a kind of valuable traditional Chinese medicine (TCM) with a history of thousands of years. The neuroprotective effects of *panax ginseng* on neurodegeneration diseases have been widely reported in the past decades, including cerebral ischemia, Alzheimer’s disease, and Parkinson’s disease (Kim et al., 2018). The key bioactive components of ginseng include more than 60 ginsenosides, such as ginsenoside Rb1, Rb2, Rb3, Rc, Rd, Re, and Rg1 (Ong et al., 2015). *In vitro* and *in vivo* studies have demonstrated that ginsenoside Rd can protect against cerebral ischemia by promoting extracellular glutamate clearance (Zhang et al., 2013). What’s more, the extracts of red ginseng could reduce cerebral infarction and edema through anti-inflammatory and anti-apoptotic pathways (Jin et al., 2022). *G. biloba* (*G. biloba* L.) is a natural medicine widely used to treat neurodegenerative diseases. The neuroprotective effects of *ginkgo biloba* on ischemic brain injury are mainly through the regulation of excitotoxicity, inflammatory pathways and oxidative damage (Feng et al., 2019). It can reduce the formation of cellular edema and neuroinflammatory injury (Zhang et al., 2018b), and regulate the imbalance of excitatory and inhibitory amino acids (Yang et al., 2011; Mdzinarishvili et al., 2012). Previous studies have shown that combining *P. ginseng* and *G. biloba* into a single treatment has a synergistic effect in clinical application, and they improve aspects of physiological and cognitive function in humans (Reay et al., 2019). Both *G. biloba* and *P. ginseng* could reduce excitotoxic insult-induced neuronal damage in the hippocampus and cortex, and the combination could improve the neuroprotective effects (Landucci et al., 2019). Interestingly, Liang et al. (2020) observed that *G. biloba* extract could enhance ginsenoside uptake by increasing the permeability of blood-brain barrier in rats. According to our previous work, the combination of *P. ginseng* and *G. biloba* extracts (CGGE) showed beneficially neuroprotective effects against neuronal apoptosis (Cong et al., 2011). What’s more, CGGE could increase acetylcholine (ACh) and improve neurodegeneration and memory deficiency (Liu et al., 2004). Besides, both *panax ginseng* and *ginkgo biloba* are the essential components of SaiLuoTong (SLT), which is an effective treatment for vascular dementia (Jia et al., 2018) to enhance cognitive and cardiovascular function (GZ et al., 2016). However, the neuroprotective effects of CGGE have not been comprehensively studied.

**FIGURE 1:**
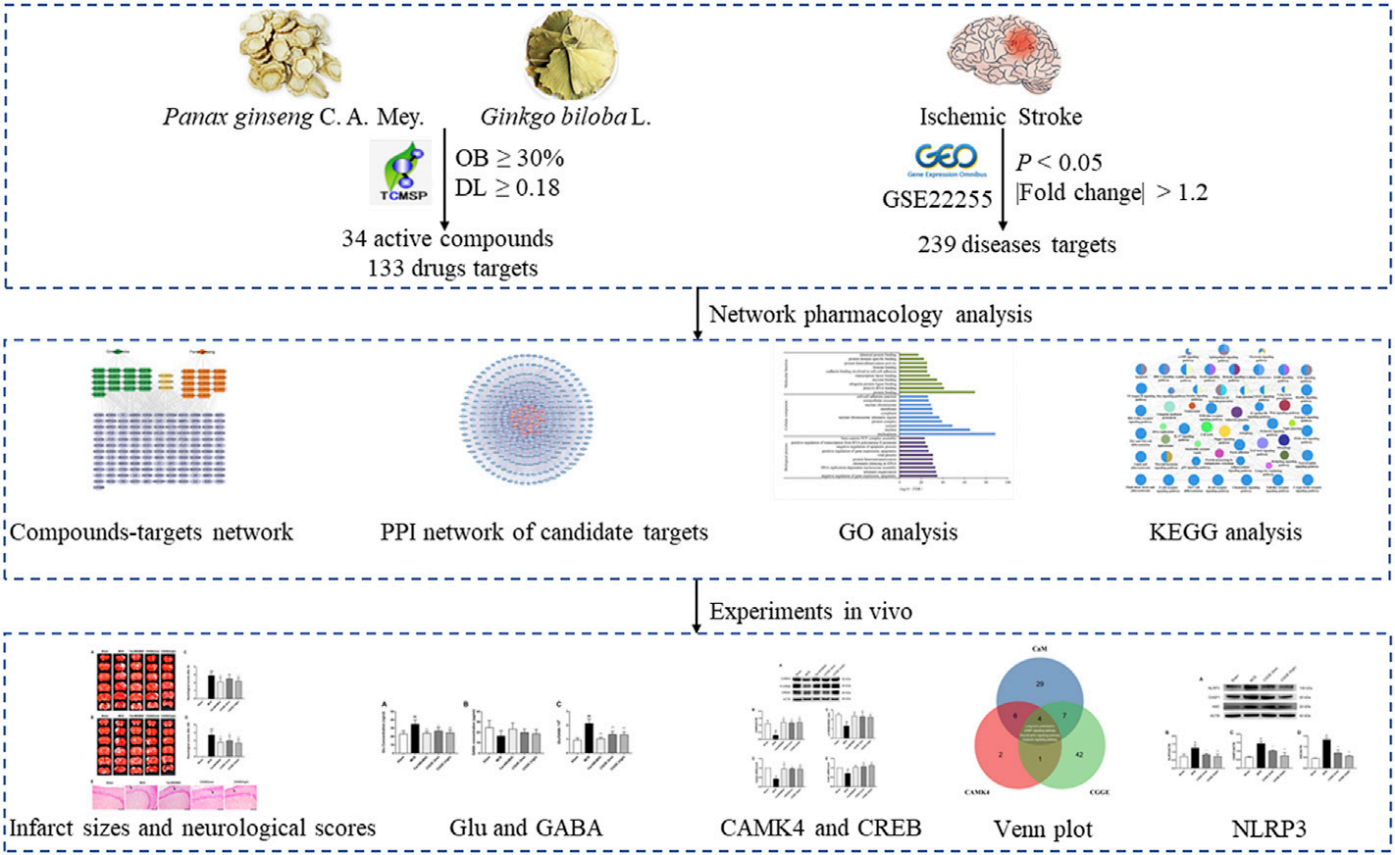
The workflow of the study.

It is more effective for predicting the therapeutic targets and mechanisms of drugs via establishing a network among the compounds, targets, and diseases utilizing various databases and tools (Zhang et al., 2019). Therefore, our present study was designed to use a network analysis approach to explore the therapeutic effects of CGGE on IS. The mechanism of CGGE ameliorating neuroinflammation and excitotoxicity with modulation of NLRP3 inflammasome and CAMK4/CREB pathway were observed in experimental research. The workflow of the study is shown in Figure1.

## 2 Materials and methods

### 2.1 Drugs and reagents

Extracts of *P. ginseng* (NO.090909) and *G. biloba* (NO.090914) were provided by the Shineway Pharmaceutical Group Co. Ltd. (Hebei, China). *Panax ginseng* extract was prepared by adding eight times water to the powder of *panax ginseng* roots and rhizomes, extracting with boiling water for 3 h, then drying with a vacuum rotary evaporator at 80°C (50 g extract from 1 kg *P. ginseng*). Ultraviolet (UV) spectroscopy or high-performance liquid chromatography (HPLC)-UV method was used to determine the extract’s total active components and main ingredients to control the quality. The quantitative analysis showed that the total ginsenoside content was 77% in ginseng extract, among which ginsenoside Rb1, Rg1, and Re are13.1% (6.55 mg/g), 5.5% (2.75 mg/g), and 3.2% (1.6 mg/g), respectively (Zhang et al., 2018c). *Ginkgo biloba* extract was obtained by adding six times volume of 70% ethanol to the powder of dry *ginkgo biloba* leaves, heating and recycling the reflux liquid, then concentration and drying (50 g extract from 1 kg *ginkgo biloba* leaves). In the extract of *ginkgo biloba*, total flavonoids accounted for 49%, quercetin, kaempferol, and isorhamnetin occupied 28.7% (14.35 mg/g), ginkgolide A, B, C and bilobalide accounted for 11.6% (5.8 mg/g), and ginkgolide A was 3.3% (1.65 mg/g) (Zhang et al., 2018c). Both ingredient extracts were mixed in a ratio of 1:1. Tat-NR2B9C, 2,3,5-triphenyltetrazolium chloride (TTC), and glutamate assay kits were obtained from Sigma (St Louis, MO, United States). GABA assay kits were purchased from USCN (Wuhan, China). Fluorescent microspheres were purchased from Sheng Yi Yao Technology Development Co., Ltd. (Beijing, China).

### 2.2 Animals

Adult male SD (Sprague-Dawley) rats (200–220 g) were provided by the Beijing Vital River Laboratory Animal Technology Co., Ltd. (License No. SCXK (Beijing) 2016-0011). All rats were housed at 25 ± 1°C temperature and 65% ± 5% humidity, with a 12 h light/dark cycle (lights on from 7:00 to 19:00). All rats had free access to food and water. The Ethics Review Committee for Animal Experimentation of Xiyuan Hospital, China Academy of Chinese Medical Sciences, approved the experimental protocols described in this study.

### 2.3 Microsphere-induced cerebral embolism rat model

Fifty rats were randomly assigned into five groups (*n* = 10): Sham group, MCE group, Tat-NR2B9C group (7.5 mg/kg), CGGE low group (15 mg/kg), and CGGE high group (30 mg/kg). The MCE rat model was established according to a previously described method (Moriyama et al., 2011). In each group, anesthesia was induced with a 2% sodium pentobarbital intraperitoneally injection (60 mg/kg). The right common carotid artery (CCA) and internal carotid artery (ICA) were occluded with vascular clamps temporarily. Immediately, a syringe with 0.2 ml serum (20 mg of the microsphere with 100–200 μm diameter was suspended in 20 ml serum) was injected into the right external carotid, and the microsphere entered the internal carotid artery from the external carotid artery. After injection, the wound was sutured and disinfected with iodophor alcohol. The rats in the sham group received the same surgical procedure without the insertion of microspheres.

CGGE was administrated before cerebral ischemia surgery and 12 h after operation by oral gavage at the dosage of 15 or 30 mg/kg. The tat-NR2B9C peptide was administered 5 min before ischemia surgery *via* the tail vein (Srejic et al., 2013). Rats in the sham group received the same volume of saline.

### 2.4 Neurological behavior scores and 2,3,5-triphenyltetrazolium chloride staining

Behavioral changes were assessed at 2 and 24 h after the operation. Neurologic symptoms were scored according to Longa’s five-point scale (Longa et al., 1989). The scales are as follows: 0, with no neurological deficit; 1, unable to fully extend the forepaw; 2, unable to move linearly or spiral to one side; 3, falling to one side; 4, no spontaneous movement. After 24 h, the rats were anesthetized and decapitated. The brains were frozen at −20°C for 5 min, and 2 mm-thick coronal slices were acquired. Then incubated in 2% TTC at 37°C for 10 min and fixed in 4% paraformaldehyde for 30 min.

### 2.5 Hematoxylin-eosin staining

HE staining was performed 24 h after cerebral ischemia. Rats were sacrificed after deep anesthetization. Brain tissues were fixed in 4% paraformaldehyde at 4°C for 24 h, dehydrated in gradient alcohols, then embedded in paraffin, and cut into 5 µm thick sections (Zhao et al., 2021). The sections were stained with HE and assessed by a light microscope (Olympus FV1200, Tokyo, Japan).

### 2.6 Network analysis

#### 2.6.1 Active compounds and drug targets screening

The active compounds and potential targets of CGGE for treating IS were investigated based on network analysis. The Traditional Chinese Medicine Systems Pharmacology Database and Analysis Platform (TCMSP, https://old.tcmsp-e.com/tcmsp.php) was used to identify and screen the active compounds of CGGE with a restriction of oral bioavailability (OB) ≥ 30% and drug-likeness (DL) ≥ 0.18 (Ru et al., 2014). The potential targets were predicted with the TCMSP database, and the reliable targets from the DrugBank database (Wishart et al., 2018) were selected for the collection. Then, the potential targets’ gene symbols were searched in the universal protein resource (UniProt, https://www.uniprot.org/).

#### 2.6.2 Screening of disease targets

The differential expressed genes of IS patients were obtained from Gene Expression Omnibus (GEO, https://www.ncbi.nlm.nih.gov/geo/, Series: GSE22255) database. Genes with *p* value <0.05 and |Fold change| > 1.2 were considered to be of significant differential expression and IS-related targets.

#### 2.6.3 Construction of protein-protein interaction networks

Referring to our previous method (Jiang et al., 2019), PPI networks of CGGE targets and IS targets were constructed by Cytoscape 3.9.0 software plugin BisoGenet, which integrates six kinds of PPI databases. The databases include Database of Interacting Proteins (DIP™), Biomolecular Interaction Network Database (BIND), Biological General Repository for Interaction Datasets (BioGRID), IntAct Molecular Interaction Database (IntAct), Human Protein Reference Database (HPRD), and Molecular INTeraction database (MINT). Then, the two PPI networks were merged with Cytoscape software. CytoNCA plugin was used to obtain the values of Degree, Closeness, Betweenness, Network, Eigenvector, Local Average Connectivity-based method (LAC), and other parameters. The nodes with more than twice the median degree were chosen as the significant targets. Moreover, the topological characteristics were calculated through degree centrality (DC), betweenness centrality (BC), and closeness centrality (CC) to filter the key targets.

#### 2.6.4 Gene ontology and pathway enrichment analysis

The Database for Annotation, Visualization, and Integrated Discovery (DAVID, https://david.ncifcrf.gov/) was used for GO enrichment analysis, and plugin ClueGO of Cytoscape 3.9.0 software was used to perform Kyoto Encyclopedia of Genes and Genomes (KEGG) pathway analysis. The biological process, cellular component, and molecular function analysis were selected for the bar charts. Pathways with significant changes of *p* < 0.05 were identified for further analysis.

### 2.7 Glu and gamma-aminobutyric acid assays

The rats were sacrificed 24 h after cerebral embolism, and the whole brain tissues were removed rapidly. Brain tissues were homogenized and centrifuged based on the product instructions. The contents of Glu and GABA in brain tissue were determined by enzyme-linked immunosorbent assay (ELISA) kits. The results were expressed as the means ± standard deviation.

### 2.8 Western blotting

The protein concentration was calculated by using bicinchoninic acid (BCA) assay kit (KGI Biotechnology Co. Ltd. China). Identical quantities of protein samples were denatured by protein loading buffer, separated by sodium dodecyl sulfate(SDS)-polyacrylamide gel electrophoresis, then transferred to PVDF membranes (Millipore, Billerica, MA, United States). The PVDF membranes were sealed with 5% bovine serum albumin (BSA) at room temperature for one hour. The following antibodies were incubated at 4°C overnight: anti-CAMK4 (1:1,000, A5304, ABclonal), anti-p-CREB (1:1,000, ab119711, Abcam), anti-CREB1 (1:1,000, A0011, ABclonal), anti-NLRP3 (1:500, DF7438, Affinity Biosciences), anti-ASC (1:500, DF6304, Affinity Biosciences), anti-CASP1 (1:500, AF5418, Affinity Biosciences). The stained blots were detected using an ECL reagent (Thermo Fisher Scientific, MA, United States). Each blot was quantified using Bio-Rad Image Lab™ Version 3.0 software. All the experiments reported in this study were performed three times, and the results were reproducible.

### 2.9 Real-time quantitative polymerase chain reaction

According to the instructions, total RNA was extracted from the brain tissue using Trizol reagent (Thermo Scientific, United States). The concentration and purity of RNA were determined by a NanoDrop 2000 spectrophotometer (Thermo Scientific, United States). The absorbance ratio (A260/280) of all samples ranged from 1.8 to 2.0, and 2 μg of total RNA was reverse transcribed into cDNA using Prime Script RT Master Mix (Takara, Dalian, China). RT-qPCR was determined using the QuantiFast^®^ SYBR^®^ Green PCR Master Mix (Qiagen, Germany) with specific primers and expression of each sample in Light Cycler^®^480ⅡReal-time PCR Instrument (Roche, Swiss), which internally normalized against Actb. The PCR primers for Camk4, Creb1, and Actb are as follows: Camk4 forward: 5′-CAC​AGA​AAT​CAG​CCT​GGT​T-3′ and reverse: 5′- ATC​TGC​TTC​ACA​GCA​TCG​C-3′; Creb1 forward: 5′- TTC​TAG​TGC​CCA​GCA​ACC-3′ and reverse: 5′-GAG​GAC​GCC​ATA​ACA​ACT​C-3′; Actb forward: 5′- CCA​CCA​TGT​ACC​CAG​GCA​TT-3′ and reverse: 5′- CGG​ACT​CAT​CGT​ACT​CCT​GC-3′. Relative expression fold change was performed by using the 2^−△△Ct^ method. Each experiment was repeated three times.

### 2.10 Statistical analysis

The results were expressed as the mean ± standard deviation (SD). The statistical analyses were performed using Statistical Package for the Social Sciences (SPSS) software (version 18.0, IBM, Chicago, IL, United States). One-way analysis of variance (ANOVA) was used to compare all groups’ differences. *p* < 0.05 was considered statistically significant.

## 3 Results

### 3.1 Combination of *panax ginseng* and *ginkgo biloba* extracts ameliorated MCE-induced neurological deficits

**FIGURE 2:**
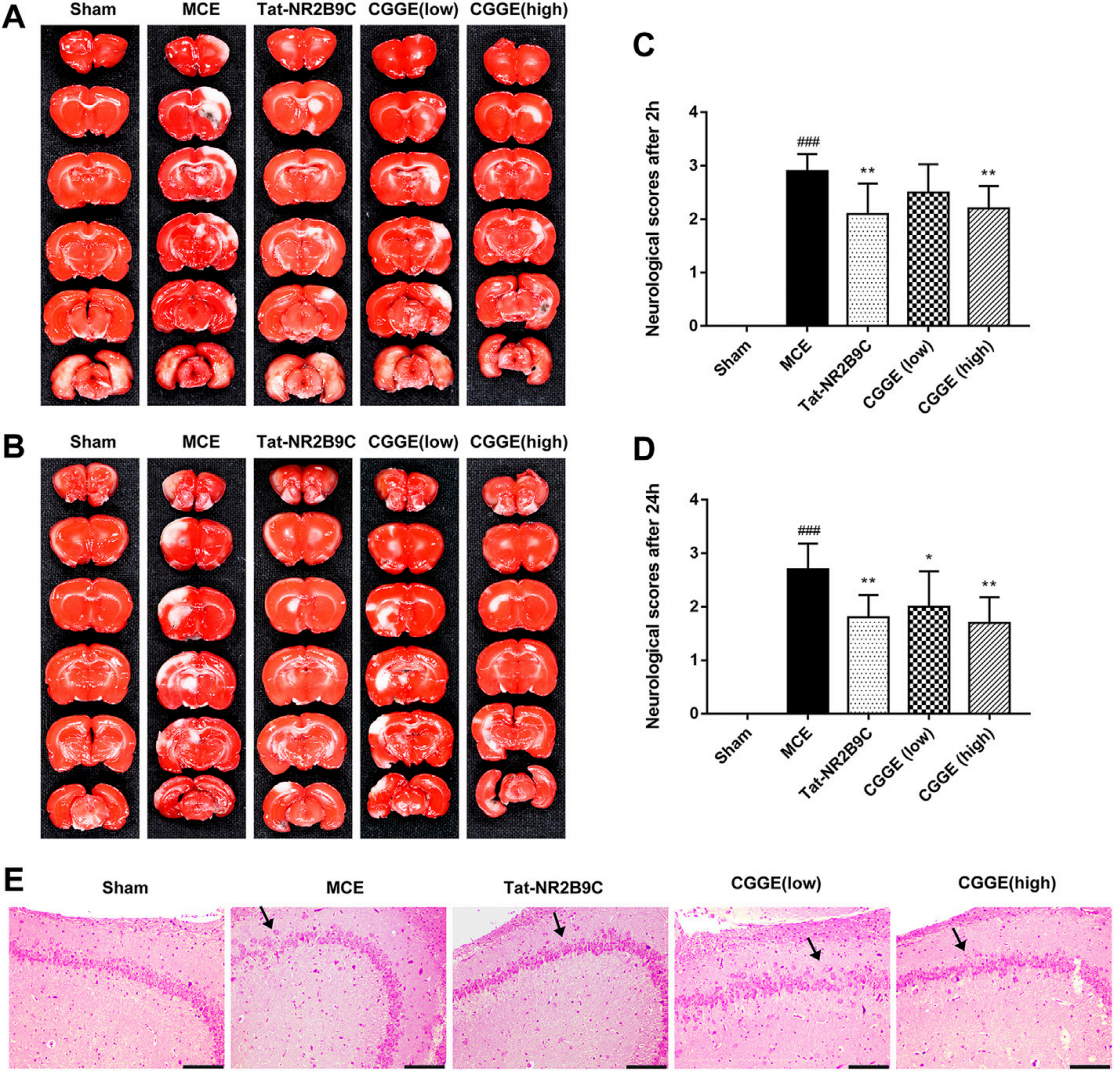
CGGE ameliorated MCE-induced neurological deficits. **(A,B)** TTC staining. The positive side **(A)** and the negative side **(B)** of the brain slices. White color indicates “infarcted area.” **(C,D)** Neurological deficit scores after 2 h **(C)** and 24 h **(D)**. All data were expressed as mean ± SD, *n* = 10, ^###^ *p* < 0.001 vs. Control group, **p < 0.01, *p < 0.05 vs. Model group. **(E)** HE staining of the hippocampus, scale bar, 200 μm (CA3 × 100).

The infarct volume of rats and neurological scores were determined after the acute treatment of the CGGE. As shown in Figures 2A,B, TTC staining results revealed that the infarct volume in the MCE group was larger than that of the sham group. However, the ischemic lesions in the Tat-NR2B9C and CGGE treatment groups were smaller than in the MCE group.

Figures 2C,D show that the neurological score of the MCE group was significantly higher than that of the sham group. Compared to the MCE group, the Tat-NR2B9C group and the high dosage CGGE group had significantly decreased scores (*p* < 0.01), while the low dosage of CGGE group could only decrease the scores after 24 h (*p* < 0.05).

Figure 2Eshows the histological changes of brain neurons with HE staining. HE stained hippocampal area (CA3) showed neuron loss, brain edema, and cell swelling. Several apoptotic neurons, such as nuclear pyknosis, intercellular space, and debris, were observed after the operation. Therefore, these results suggested that CGGE could attenuate brain injury induced by cerebral ischemia with a dose-dependent.

### 3.2 Compound-target network analysis

**FIGURE 3:**
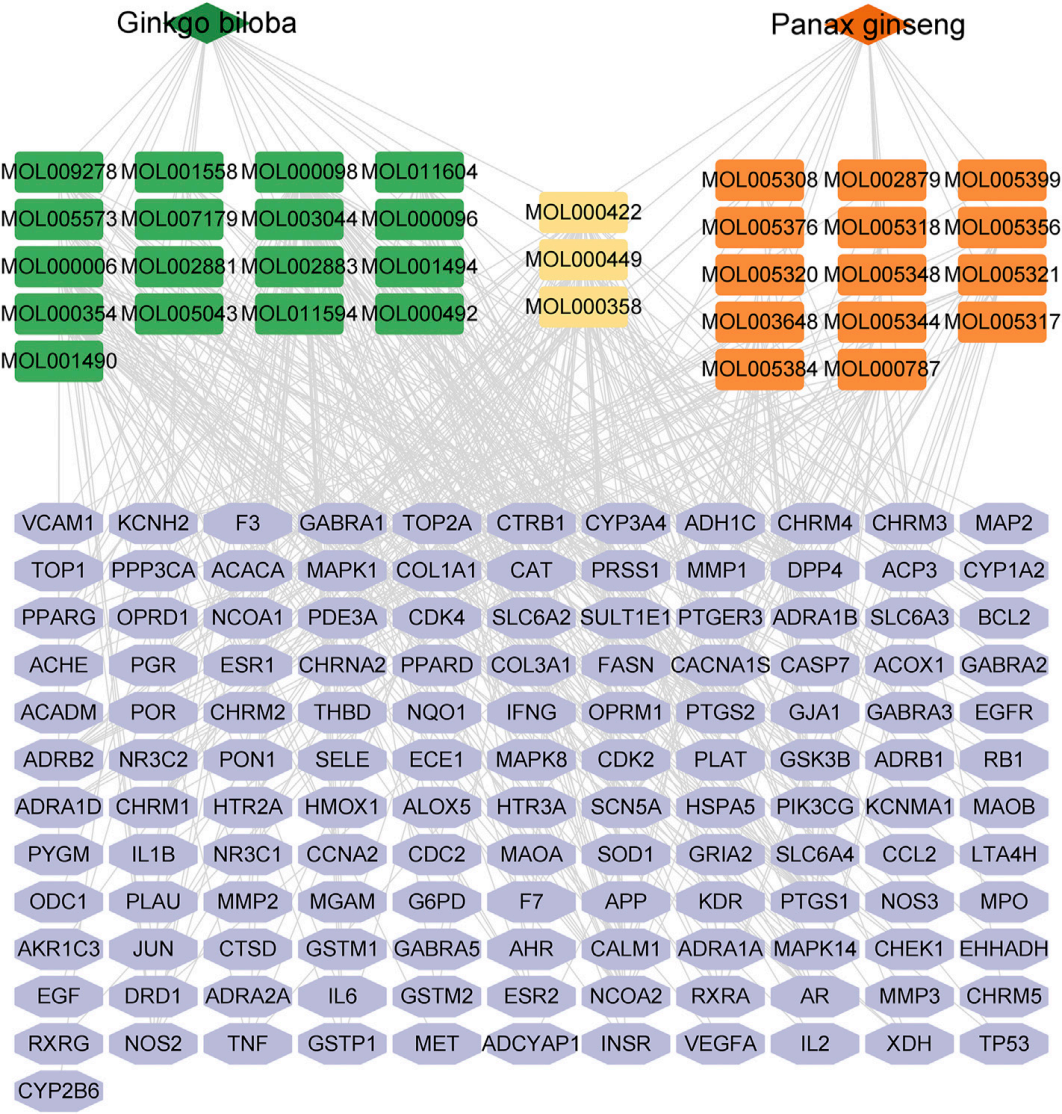
The compound-target network of CGGE.

The network analysis approach was used to elucidate the active components and mechanisms of the CGGE against IS. The chemical constituents of CGGE were obtained from the TCMSP database (Version: 2.3) with screening parameters of OB ≥ 30% and DL ≥ 0.18. Twenty-two compounds of *Panax ginseng* and 27 compounds of *Ginkgo biloba* were obtained. A total of 34 components were characterized after removing the compounds without targets and repetitions (Table 1). The total number of potential targets for 34 compounds was 133. The compound-target network of CGGE is shown in Figure 3 **.**

**TABLE 1:** The chemical compositions of CGGE.

Mol Id	Chemical compositions	OB(%)	DL	Source
MOL002879	Diop	43.59	0.39	*Panax ginseng*
MOL003648	Inermin	65.83	0.54	*Panax ginseng*
MOL005308	Aposiopolamine	66.65	0.22	*Panax ginseng*
MOL005317	Deoxyharringtonine	39.27	0.81	*Panax ginseng*
MOL005318	Dianthramine	40.45	0.2	*Panax ginseng*
MOL005320	Arachidonate	45.57	0.2	*Panax ginseng*
MOL005321	Frutinone A	65.9	0.34	*Panax ginseng*
MOL005344	Ginsenoside rh2	36.32	0.56	*Panax ginseng*
MOL005348	Ginsenoside-Rh4_qt	31.11	0.78	*Panax ginseng*
MOL005356	Girinimbin	61.22	0.31	*Panax ginseng*
MOL005376	Panaxadiol	33.09	0.79	*Panax ginseng*
MOL005384	Suchilactone	57.52	0.56	*Panax ginseng*
MOL005399	Alexandrin_qt	36.91	0.75	*Panax ginseng*
MOL000787	Fumarine	59.26	0.83	*Panax ginseng*
MOL011594	Isogoycyrol	40.36	0.83	*Ginkgo biloba*
MOL011604	Syringetin	36.82	0.37	*Ginkgo biloba*
MOL001490	Bis [(2 S)-2-ethylhexyl]benzene-1,2-dicarboxylate	43.59	0.35	*Ginkgo biloba*
MOL001494	Mandenol	42	0.19	*Ginkgo biloba*
MOL001558	Sesamin	56.55	0.83	*Ginkgo biloba*
MOL002881	Diosmetin	31.14	0.27	*Ginkgo biloba*
MOL003044	Chryseriol	35.85	0.27	*Ginkgo biloba*
MOL000354	Isorhamnetin	49.6	0.31	*Ginkgo biloba*
MOL000492	(+)-Catechin	54.83	0.24	*Ginkgo biloba*
MOL005573	Genkwanin	37.13	0.24	*Ginkgo biloba*
MOL000006	Luteolin	36.16	0.25	*Ginkgo biloba*
MOL007179	Linolenic acid ethyl ester	46.1	0.2	*Ginkgo biloba*
MOL009278	Laricitrin	35.38	0.34	*Ginkgo biloba*
MOL000096	(−)-Catechin	49.68	0.24	*Ginkgo biloba*
MOL000098	Quercetin	46.43	0.28	*Ginkgo biloba*
MOL002883	Ethyl oleate (NF)	32.4	0.19	*Ginkgo biloba*
MOL005043	Campest-5-en-3beta-ol	37.58	0.71	*Ginkgo biloba*
MOL000449	Stigmasterol	43.83	0.76	*Panax ginseng*/*Ginkgo biloba*
MOL000358	Beta-sitosterol	36.91	0.75	*Panax ginseng*/*Ginkgo biloba*
MOL000422	Kaempferol	41.88	0.24	*Panax ginseng*/*Ginkgo biloba*

The network contained 169 nodes and 468 edges which indicated the compound-target interactions. The median degree of 34 chemical components is 9, indicating that compounds could interact with multiple targets. The information on the top 10 chemical components and targets is shown in Table 2. The higher the degree, the more important the compound was in the network. According to the results, the important chemical compounds of CGGE included quercetin, kaempferol, beta-stigmasterol, stigmasterol, luteolin, and the crucial targets of CGGE were PTGS2, NCOA2, PTGS1, CALM1, DPP4, and SCN5A.

**TABLE 2:** The main chemical compositions and targets of CGGE.

NO.	Composition	degree	Target	degree
1	Quercetin	70	PTGS2	25
2	Kaempferol	43	PTGS1	22
3	Beta-sitosterol	30	NCOA2	18
4	Stigmasterol	29	CALM1	12
5	Luteolin	29	DPP4	12
6	Isorhamnetin	28	SCN5A	12
7	Fumarine	25	AR	11
8	Syringetin	18	ADRB2	10
9	Laricitrin	18	PIK3CG	10
10	Chryseriol	17	PRSS1	10

### 3.3 Identification of candidate targets for combination of *panax ginseng* and *ginkgo biloba* extracts against ischemic stroke

The reliability of the test results can be significantly improved by directly selecting the human chip results and setting relevant screening conditions for analysis. The GEO database identified two hundred and thirty-nine IS-related targets (Series: GSE22255).

**FIGURE 4:**
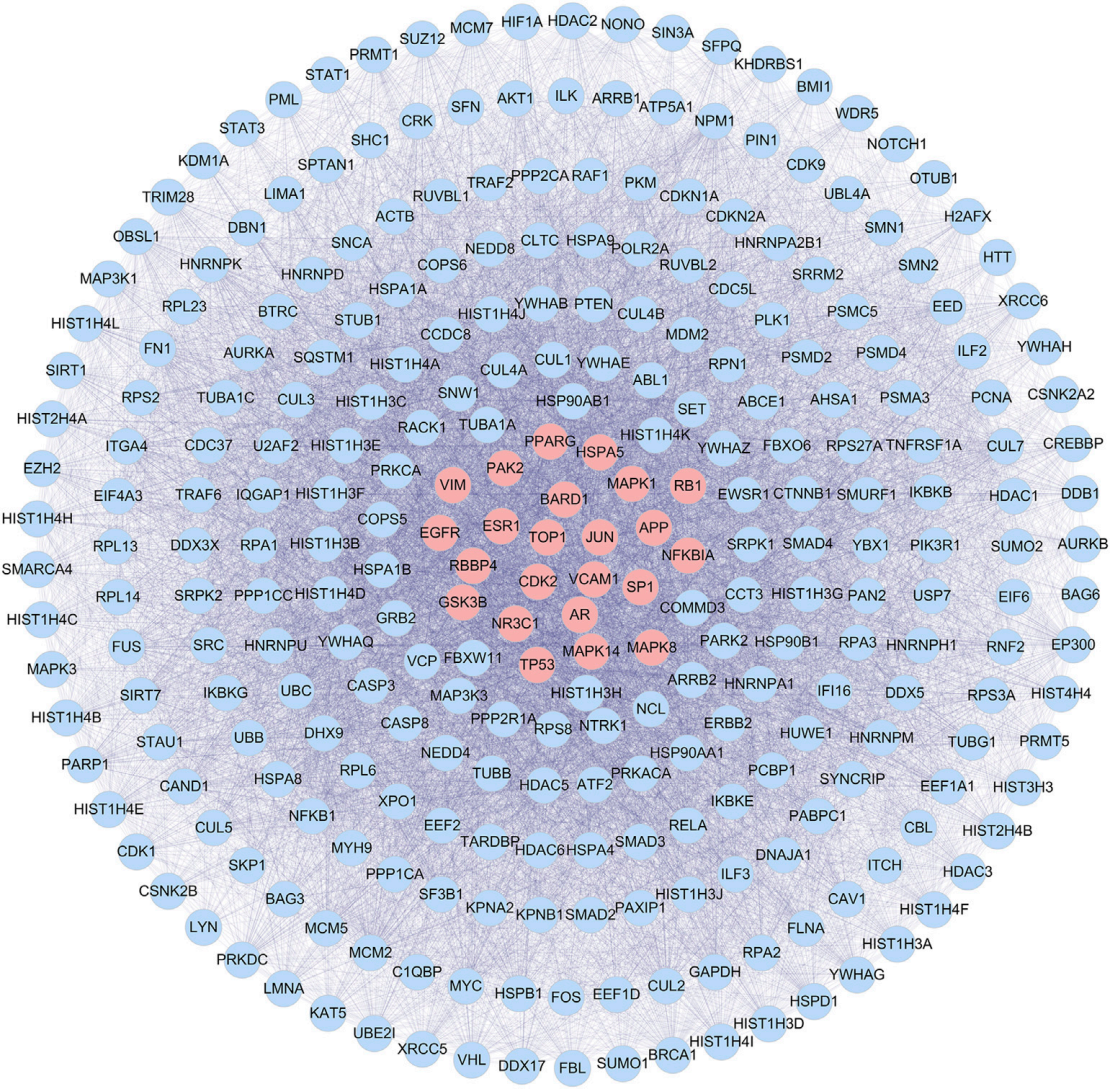
PPI network of candidate targets of CGGE against IS.

CGGE targets PPI network and IS-related targets PPI network were constructed. 6,275 CGGE target proteins and 5,259 IS-related target proteins were obtained (Supplementary Tables S1, S2). In order to reveal the relationship between CGGE and IS, the PPI networks of CGGE targets and IS-related targets were merged to identify the candidate targets for CGGE against IS. The network consists of 3,494 proteins (Supplementary Tables S3). The median degree of all nodes was 35, and the nodes with more than 70° were identified as significant targets. A network of significant targets for CGGE against IS was constructed, containing 848 proteins (Supplementary Table S4). The common targets were the critical goal of CGGE treatment in IS and the vital targets screened for further research. Subsequently, the topological feature values of common targets in the network, including DC, BC, and CC, were analyzed for the important key targets. Finally, 288 nodes were chosen as the candidate targets with DC > 112.5, BC > 6,455.9, and CC > 0.467 **(** Figure 4 **)**.

### 3.4 Gene ontology and Kyoto Encyclopedia of Genes and Genomes pathway enrichment analysis

**FIGURE 5:**
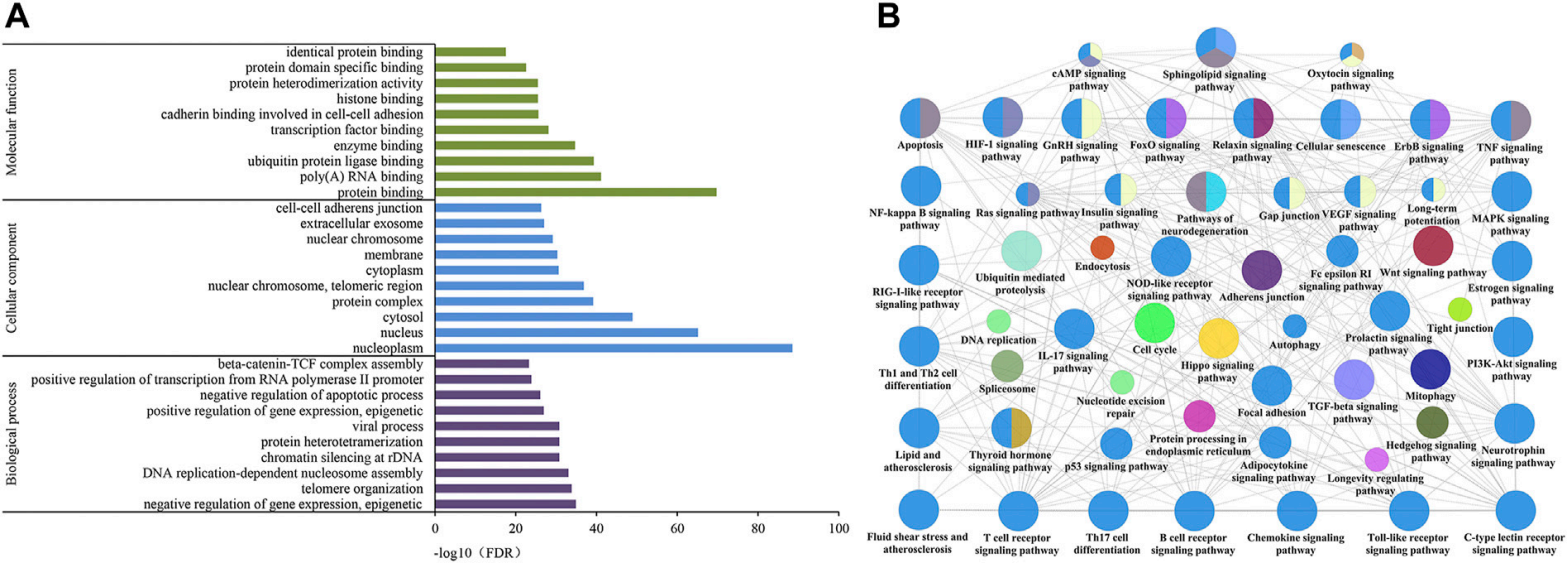
GO **(A)** and KEGG **(B)** pathway enrichment analysis of CGGE against IS.

DAVID was used to perform GO analysis of the 288 identified candidate targets. GO of candidate targets was analyzed based on the domains “biological process,” “cellular component,” and “molecular function.” The top 10 terms are shown in Figure 5A. The highly enriched GO terms in biological process, cellular component, and molecular function included negative regulation of gene expression, nucleoplasm, nucleus, cytosol, protein binding, and ubiquitin-protein ligase binding.

In order to further analyze the biological mechanisms involved with the 288 candidate targets, the ClueGO was used to perform the KEGG pathway analysis. As shown in Figure 5B **,** the mechanisms of CGGE against IS might be concerned with long-term potentiation, cAMP signaling pathway, neurotrophin signaling pathway, and NOD-like receptor signaling pathway.

### 3.5 Combination of *panax ginseng* and *ginkgo biloba* extracts modulated the level of Glu and GABA concentration in ipsilateral cortex

**FIGURE 6:**
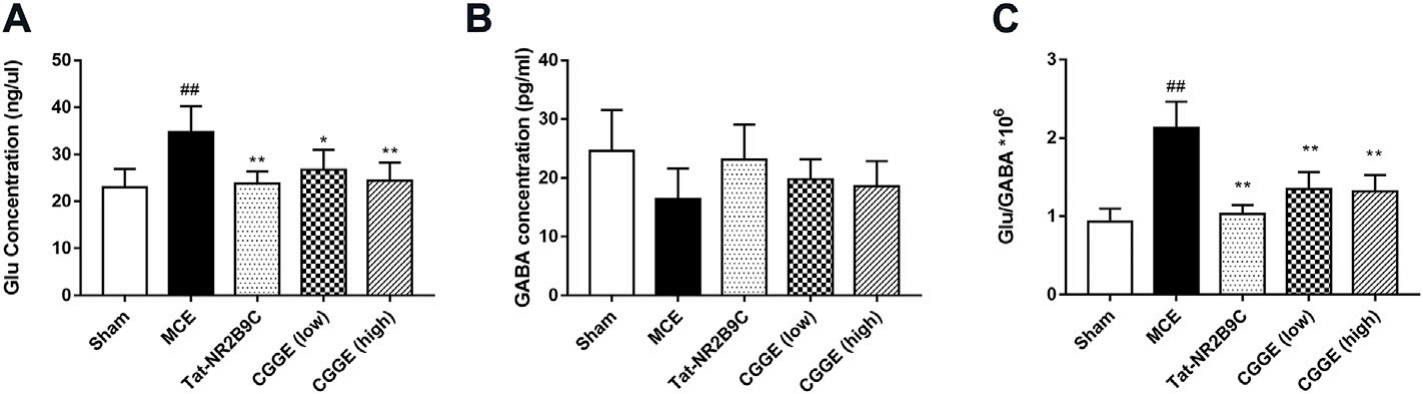
CGGE modulated the glutamate and GABA concentration level. **(A)** The changes in Glu level. **(B)** The changes in GABA level. **(C)** Changes in the Glu/GABA ratio. ^##^ *p* < 0.01 vs. sham group; **p < 0.01, *p < 0.05 vs. MCE group (*n* = 6).

The changes in Glu and GABA levels in the ipsilateral cortex were illustrated in Figure 6 (*n*= 6). It was found that the Glu level was significantly higher in the MCE group than in the sham group (*p* < 0.01) (Figure 6A). The Tat-NR2B9C and CGGE treatments significantly reduced the Glu level compared to the MCE group (*p* < 0.01, *p* < 0.05, and *p* < 0.01, respectively). However, no significant differences in the GABA level were observed among all groups (Figure 6B). The ratio of Glu/GABA (Figure 6C) was found to be higher in the MCE group than that in the sham group (*p* < 0.01). Treatment groups significantly reduced the Glu/GABA ratios compared to the MCE group (*p* < 0.01).

### 3.6 Combination of *panax ginseng* and *ginkgo biloba* extracts activated CAMK4/ CREB protein in ipsilateral cortex

**FIGURE 7:**
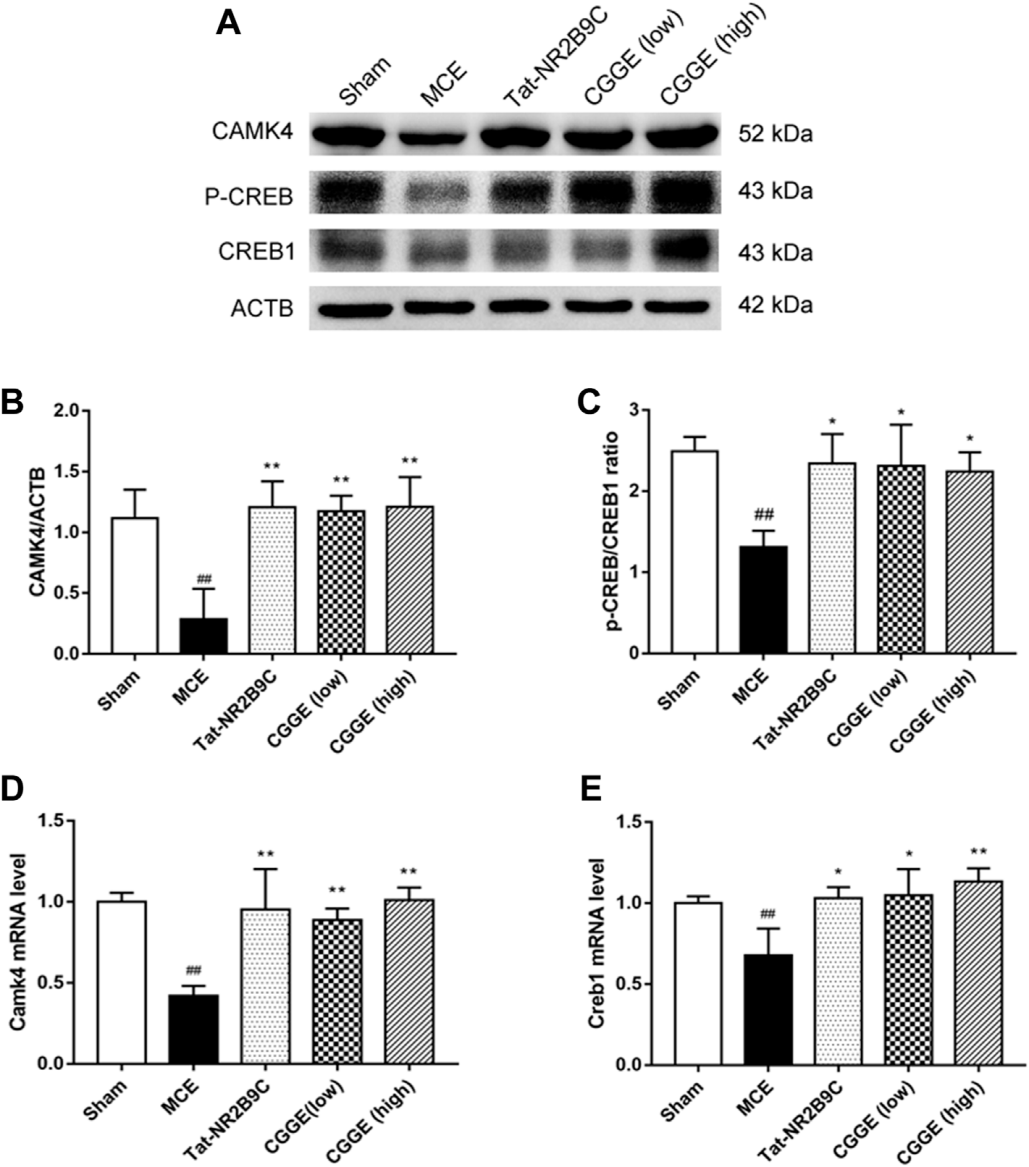
CGGE activated CaMKⅣ/CREB in the ipsilateral cortex. **(A)** Protein expression levels of CAMK4, p-CREB, CREB1. **(B,C)** The analysis of protein expression of CAMK4, p-CREB/CREB1. **(D,E)** The mRNA expression of *Camk4* and *Creb1*. (^##^ *p* < 0.01, vs. sham group; **p < 0.01, *p < 0.05, vs. MCE group) (*n* = 3).

The effects of CGGE on the CAMK4/CREB are shown in Figure 7A. The protein expression of CAMK4 and p-CREB were decreased following ischemic insult (Figures 7B,C, *p* < 0.01). CAMK4 and p-CREB expression changes were reversed following the treatments of Tat-NR2B9C and CGGE. The mRNA expression of *Camk4* and *Creb1* was decreased in the MCE group, whereas it increased with Tat-NR2B9C and the CGGE treatments (Figures 7D,E).

### 3.7 Combination of *panax ginseng* and *ginkgo biloba* extracts inhibited NLRP3 inflammasome formation in MCE rats

**FIGURE 8:**
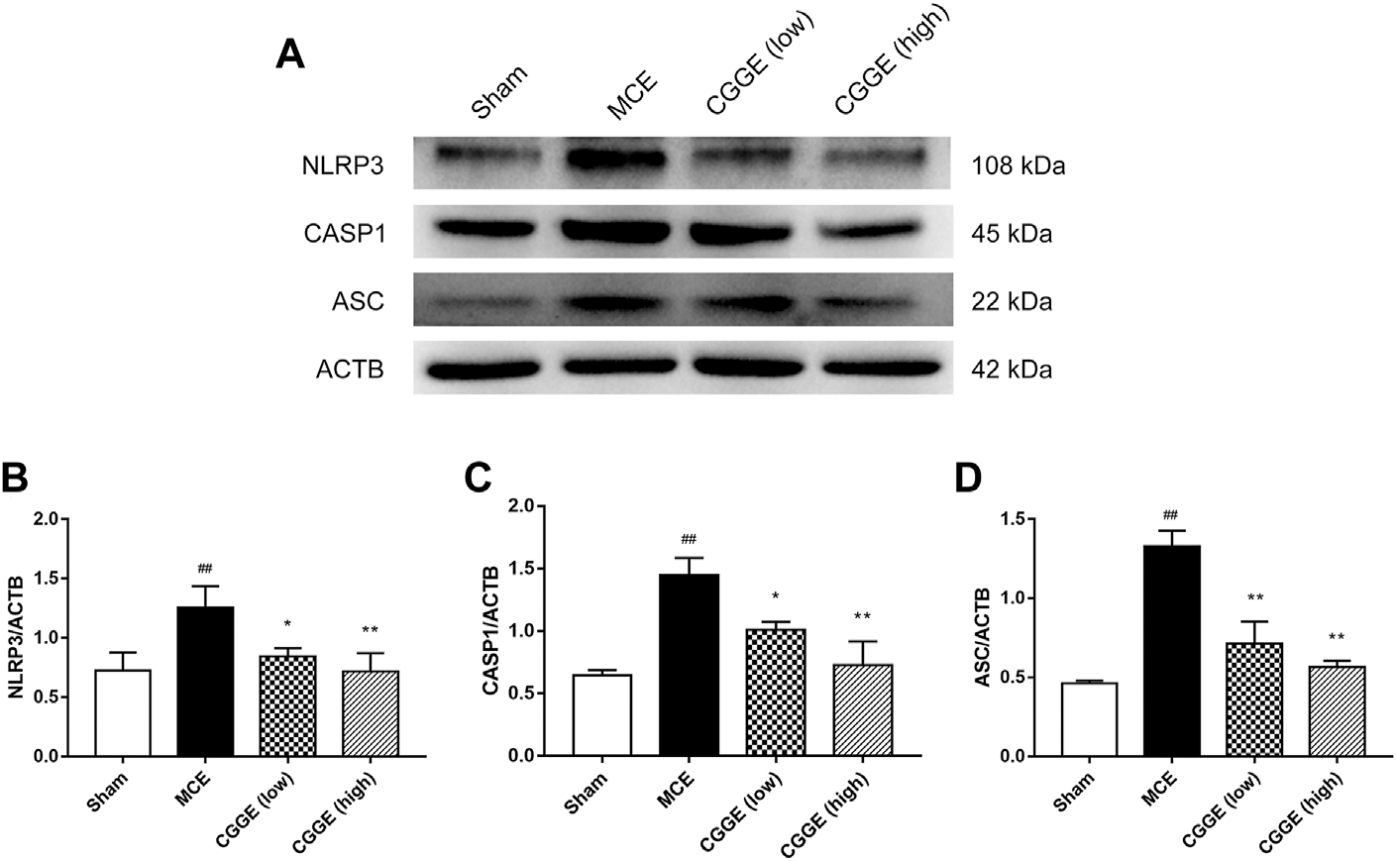
CGGE inhibited NLRP3 Inflammasome in ipsilateral cortex. **(A)** Protein expression of NLRP3, CASP1, and ASC. **(B,C,D)** The analysis of protein expression of NLRP3, CASP1, and ASC. (^##^ *p* < 0.01, vs. sham group; **p < 0.01, *p < 0.05, vs. MCE group) (*n* = 3).

To investigate the potential mechanism by which CGGE alleviates neuroinflammation, we examined the expression of NLRP3 inflammasome in the ipsilateral cortex (*n* = 3). Results are shown in Figure 8. The protein expression of NLRP3, ASC, and CASP1 were significantly increased after cerebral ischemia (*p* < 0.01). Compared to the MCE group, CGGE significantly decreased the expression of NLRP3, ASC, and CASP1 proteins. In addition, CGGE exhibited concentration-dependent inhibition of the protein expression.

## 4 Discussion

The present study demonstrated that CGGE could reduce the infarct size and neurological deficits via alleviating excitotoxicity and neuroinflammation in MCE rats. Glu and GABA are the central nervous system’s principal excitatory and inhibitory neurotransmitters. Excessive glutamate overstimulates the ionic glutamate receptor and causes excessive calcium influx, resulting in excitotoxicity to neurons, which is one of the major reasons for neuronal dysfunction and degeneration (Lau and Tymianski, 2010). The results revealed that CGGE could maintain glutamate and GABA balance via activating CAMK4/CREB pathway, which plays a vital role in maintaining normal neural function and reducing secondary brain injury. In addition, CGGE also showed an anti-inflammation effect by inhibiting the activity of NLRP3 inflammasome.

Network pharmacology analysis is a new method that has been applied to predict the pharmacological mechanism of TCM (Li and Zhang, 2013). The potential mechanisms of CGGE against IS-mediated neurotoxicity and neuroinflammation were explored using a network analysis approach. Thirty-four active compounds of CGGE and 133 targets were used to construct a compound-target network. Results suggested that PTGS2, PTGS1, NCOA2, CALM1, DPP4, and SCN5A might be the critical targets of CGGE. CALM1 is the encoding gene of calmodulin (CaM). CaM is related to the calcium signal transduction pathways and is crucial in controlling cellular functions throughout all stages (O'Day et al., 2020). Therefore, CaM might be the key target of CGGE for treating IS. CaM is a Ca^2+^ multifunctional binding protein. Ca^2+^/CaM binding modulates various proteins, including CAMKs (Cohen et al., 2016) and cAMP (Lukyanenko et al., 2016). Ca^2+^ influx triggers phosphorylation of CREB, which binds to a critical Ca^2+^ response element (CRE) (Tao et al., 1998). CRE could activate the transcription of BDNF. The release of BDNF stimulates TrkB receptors on GABAergic interneurons and increases the input of GABAergic neural precursors, thus stimulating their differentiation and maturation into neurons to balance the excitotoxicity of glutamate (Waterhouse et al., 2012). Ca^2+^/CaM activates CaM kinases (CaMKs) and CREB kinases from the surface membrane to the nucleus, which is important for synaptic potentiation, learning, and memory (Zhang et al., 2016). In various CaMKs, CaMK4 is thought to phosphorylate CREB in the nuclei (Impey et al., 2002). Interestingly, the results suggested that CGGE may play a neuroprotective role by activating CaMK4 and CREB.

**FIGURE 9:**
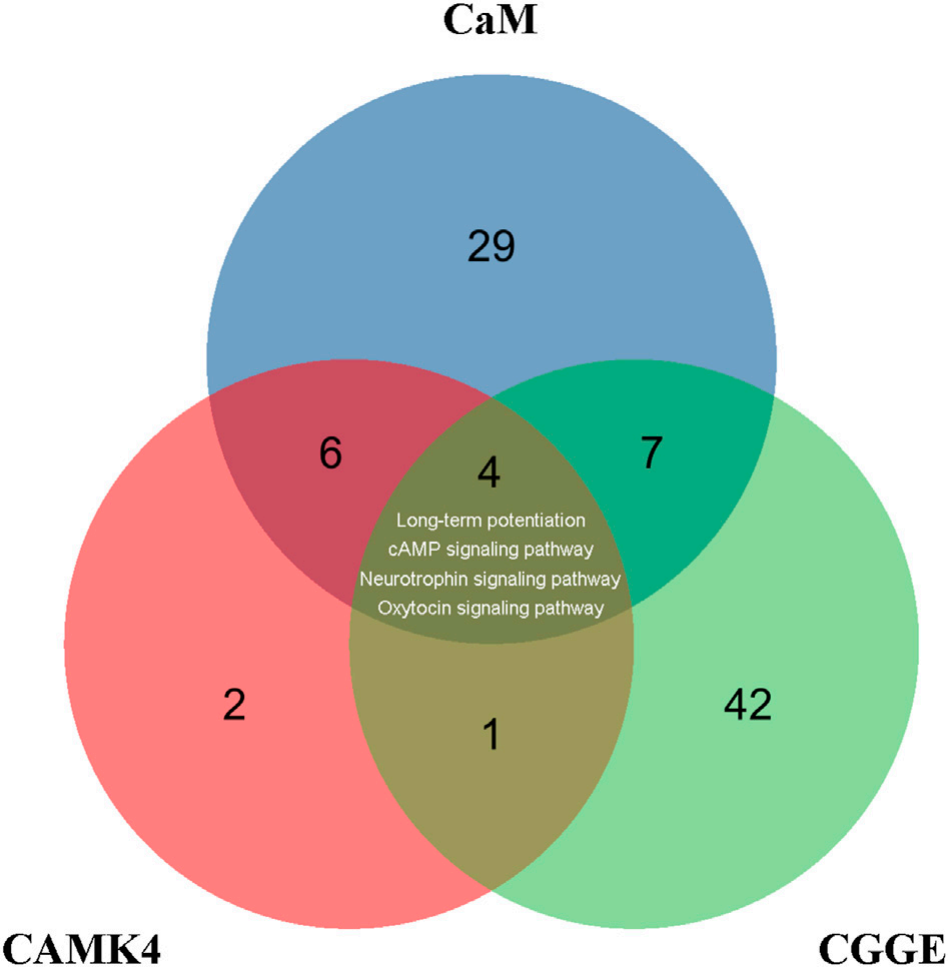
Venn diagram depicts the overlap of CaM, CAMK4, and CGGE-involved signaling pathways. Long-term potentiation, cAMP signaling pathway, and neurotrophin signaling pathway are related to the activation of CREB.

CREB is a crucial transcription factor, and the inactivation of CREB leads to the inhibition of many CREs promoters, including neurotrophic factors and synaptic proteins. The activity of CREB is regulated by phosphorylation and dephosphorylation, and many protein kinases can phosphorylate CREB, such as protein kinase A (PKA) (Dash et al., 1991), mitogen-activated protein kinase (MAPK) (Xing et al., 1996), phosphoinositide-3-kinase (PI3K)-Akt (Zhang W. et al., 2018), and CaMKs (Sheng et al., 1991). CaM is involved in 43 signaling pathways in the KEGG database, while CaMKⅣ is associated with 13 signaling pathways (Figure 9). There were ten common signaling pathways for CaM and CAMK4: long-term potentiation, cAMP signaling pathway, neurotrophin signaling pathway, and calcium signaling pathway. Accordingly, the CGGE targets PPI network and IS-related targets PPI network were structured and merged, and 288 targets were finally identified to elucidate the underlying neuroprotective mechanisms of CGGE. In the KEGG pathway enrichment analysis, long-term potentiation, cAMP signaling pathway, and neurotrophin signaling pathway are closely related to the activation of CREB. The results showed that CGGE might play a role in treating IS by activating CaM and CREB through long-term potentiation, cAMP signaling pathway, and neurotrophin signaling pathway.

Inflammation plays a key role in the pathophysiology of ischemic stroke. NLRP3 inflammasome is the best-characterized inflammasome and one of the major contributors to neuroinflammation, which consists of NLRP3, ASC, and Caspase-1 (Alishahi et al., 2019). The KEGG pathway enrichment analysis showed that the NOD-like receptor signaling pathway was the potential mechanism of CGGE against IS. After cerebral ischemia, the changes in the intracellular microenvironment trigger the NLRP3 inflammasome (NLRP3/ASC/CASPASE-1) activation, which leads to the secretion of IL-1β and IL-18 and finally mediated cell death. Western blot analysis showed that CGGE could downregulate the protein expression of NLRP3 in the ipsilateral cortex. The data suggested that CGGE exerted an anti-inflammatory effect after ischemic stroke by inhibiting NLRP3 activation.

Taken as a whole, the present data suggested that the neuroprotective mechanism of CGGE against IS was related to excitotoxicity and neuroinflammation, which mainly through the long-term potentiation, cAMP signaling pathway, neurotrophin signaling pathway, and NOD-like receptor signaling pathway. However, there are still some limitations. Firstly, the rat model of cerebral ischemia was established by microsphere injection, which prevented an accurate assessment of the infarct sizes. Secondly, we only measured the content of amino acids 24 h after ischemic injury, while the change in amino acid level is a continuous process. More sampling points, especially during the early stage of ischemia, would help better understand the dynamic changes of the neurotransmitters and the related mechanisms. Therefore, further studies are expected to explore the deeper mechanism.

## 5 Conclusion

In conclusion, the underlying mechanisms of CGGE on IS were explored via the integration of network analysis and *in vivo* experiments. The results showed that CGGE could improve neurological function via alleviating excitotoxicity and neuroinflammation. According to the network analysis, CALM1 might be the critical target of CGGE. Furthermore, the protective mechanisms of CGGE on ischemic stroke might involve long-term potentiation, cAMP signaling pathway, neurotrophin signaling pathway, and NOD-like receptor signaling pathway. The mechanisms by which CGGE exerts anti-inflammatory and anti-excitotoxicity effects involved the inhibition of NLRP3 inflammasome and upregulation of the CAMK4/CREB pathway.

## Data Availability

The datasets presented in this study can be found in online repositories. The names of the repository/repositories and accession number(s) can be found in the article/Supplementary Material.

## Glossary

CGGE: combination of panax ginseng and ginkgo biloba extracts
TCM: traditional chinese medicine
IS: ischemic stroke
MCE: microsphere-induced cerebral embolism
TTC: 2,3,5-triphenyltetrazolium chloride
HE: hematoxylin-eosin
GABA: gamma-aminobutyric acid
Glu: glutamate
ELISA: enzyme linked immunosorbent assay
RT-qPCR: real-time quantitative polymerase chain reaction
BCA: bicinchoninic acid
NMDAR: N-methyl-D-aspartate receptor
PSD-95: postsynaptic density-95
SDS: sodium dodecyl sulfate
MAPK: mitogen-activated protein kinase
CaMKs: calmodulin-dependent protein kinases
CaMKⅣ: calmodulin-dependent protein kinase type Ⅳ
NLRP3: Nod-like receptor, pyrin containing 3
PKA: protein kinase A
PI3K: phosphoinositide-3-kinase
CREB: cyclic AMP-responsive element-binding protein
BDNF: brain-derived neurotrophic factor
CCA: common carotid artery
ICA: internal carotid artery
SD: standard deviation
GEO: gene expression omnibus
PPI: protein-protein interaction
LAC: local average connectivity-based method
DC: degree centrality
BC: betweenness centrality
CC: closeness centrality
GO: gene ontology
OB: oral bioavailability
DL: drug-likeness
HPLC: high-performance liquid chromatography
UV: Ultraviolet
